# Patient safety attitudes and their predictors among emergency healthcare providers in a military medical city in the Kingdom of Saudi Arabia: a cross-sectional study

**DOI:** 10.3389/fmed.2025.1541273

**Published:** 2025-09-22

**Authors:** Adel Saeed S. Alotaibi, Salem Ali S. Alshehri, Naif H. Alanazi, Abdualrahman S. Alshehry, Homood A. Alharbi, Abdulmajeed Wadid A. Alanazi, Ali Ahmed M. Alkhalaf, Gerlynn C. Tumala, Abdulaziz Moadi B. Alotaibi, Mohammed Ahmed Alfaifi, Regie B. Tumala

**Affiliations:** ^1^Emergency Nursing Department, Prince Sultan Military Medical City, Riyadh, Saudi Arabia; ^2^College of Nursing, King Saud University, Riyadh, Saudi Arabia; ^3^Academic Affairs, Prince Sultan Military Medical City, Riyadh, Saudi Arabia; ^4^Health Education Affairs, Prince Sultan Military Medical City, Riyadh, Saudi Arabia; ^5^Nursing Department, King Abdulaziz University Hospital, Medical City, King Saud University, Riyadh, Saudi Arabia

**Keywords:** attitude, emergency department, emergency medical technician, health care provider, nurse, paramedic, patient safety, physician

## Abstract

**Background:**

The increase in the number of patient safety incidents poses a challenge for hospital management. Various studies have been conducted on the safety of patients in healthcare settings, but gaps exist concerning the attitude of healthcare providers (HCPs) toward the safety of patients, including those in the emergency department (ED) in Saudi Arabian hospitals.

**Aim:**

This study aimed to determine the attitude of HCPs toward patient safety in the ED at Prince Sultan Military Medical City (PSMMC) and to identify demographic factors predictive of HCPs’ attitude.

**Methods:**

A descriptive–correlational design was used. The study was conducted on a convenience sample of 202 HCPs working in the ED at PSMMC. Data were collected in December of 2022 and analyzed using SPSS v.23. Multiple linear regression analyses were performed separately for the six domains and overall patient safety attitude as dependent variables. For the seven models, the demographic variables were considered predictor variables.

**Results:**

Overall, HCPs demonstrated a positive attitude toward patient safety (Mean = 3.75/5). Among the six domains, job satisfaction scored highest (Mean = 3.96), while stress recognition scored lowest (Mean = 3.54). However, the respondents reported some patient safety issues and agreed on the difficulty in speaking up if they perceived a problem with patient care, as well as in discussing errors. Significant relationships and predictors were established in all six dimensions and in the overall patient safety attitude. The study findings revealed that three demographic variables, namely, certification in ED (*p* = 0.044), value of patient safety (*p* = 0.001), and profession (*p* = 0.014), demonstrated significant correlations with the overall attitude toward patient safety. Furthermore, years of experience as an HCP in ED (*p* = 0.019), value of patient safety (*p* = 0.004), and profession (*p* = 0.047) were identified as predictors of the overall patient safety attitude.

**Conclusion:**

The overall attitude of HCPs toward patient safety in the ED at PSMMC was found to be positive across all six domains and overall. Several demographic factors were identified as significantly influencing their positive attitudes toward patient safety. However, some patient safety issues still need to be resolved, needing interventional and strategic solutions from hospital administration. These solutions should take into account, and give high consideration to, the significant demographic factors identified as predictors of HCPs’ attitudes toward patient safety.

## 1 Introduction

The culture of an organization is a significant part of the healthcare environment because it influences not only the procedures carried out by healthcare providers (HCPs) but also their perceptions, considerations, and attitudes when interacting with patients (1, 2). The safety culture of an organization has various components, including HCPs’ attitudes toward staff-related factors (such as excessive self-assurance and overconfidence), team-related factors (such as supervision and teamwork), work environment factors (such as managerial support and staffing levels), and organizational factors (such as morale and safety climate). Research has identified safety attitude as a critical part of the culture of an organization (3, 4). This involves the culture of safety within an organization that could be defined as values, competencies, perceptions, beliefs, and attitudes that inform the management of the safety and health of the organization as embraced by employees with regard to the safety of the patients (1). An understanding of the expectations and perceptions of HCPs toward adverse events is critical to implement proper strategies for managing patient care. Conducting such a study in the emergency department (ED) of Prince Sultan Military Medical City (PSMMC) is important because of the high number of patients being seen and the high number of incidents. In addition, minimal research has been conducted with regard to the safety attitude of HCPs in hospitals across Saudi Arabia.

Patient safety is important in hospital settings and a part of the organizational culture of healthcare organizations (5, 6). Critical patient safety and health issues occur within the healthcare setting on a daily basis. Such issues have detrimental consequences, ranging from financial costs to irreversible disabilities, prolonged hospitalizations, and patient deaths. Similar to healthcare organizations worldwide, Saudi Arabian hospitals have been linked to negative healthcare and patient health outcomes as a result of patient safety issues (7). Aljarallah and Alrowaiss (8) examined 642 adverse events in hospitals in Saudi Arabia, 18.1% of which were linked to EDs, while 20.4% occurred in operating rooms. Such is a clear indication of the significance of maintaining a safety climate in the ED and the implications of HCP involvement in promoting such safety.

According to Alharbi, Cleland, and Morrison (9), the safety climate of a hospital and the safety attitude of HCPs are among the most significant factors that influence the rates of errors and the quality of care received by patients. Alzahrani, Jones, and Abdel-Latif (10) evaluated the attitude of nurses and doctors toward the safety of patients in the EDs of two hospitals in Saudi Arabia. They established that nurses and doctors had a less positive attitude toward the safety of patients in the ED (10). The findings have a positive correlation with the high number of errors reported by the departments. These findings are comparable to those of Algahtani (11), who established that the negative safety attitude of nurses contributed to the high incidence rate of medical errors. Alayed, Loof, and Johansson (7) reported similar findings in their examination of the attitudes of nurses in the intensive care units (ICUs) of six Saudi hospitals, whereby nurses had a negative attitude toward patient safety. Alonazi et al. (12) also determined how nurses perceived the safety culture and established that safety attitude among them were sub-optimal, emphasizing the need for improving the organization’s safety culture. Overall, the attitude of HCPs toward patient safety is critical to effectively facilitate positive patient outcomes within the healthcare environment.

Various studies are available with regard to the safety of patients in healthcare settings, but gaps exist concerning the attitude of HCPs toward the safety of patients in the ED of Saudi Arabian hospitals. Minimal research has been conducted, and evidence on the safety attitude of HCPs in hospitals in Saudi Arabia is limited, which is an integral area of consideration given the high-risk setting of EDs. Limited evidence is also available in comparing the implications of the attitude of nurses and physicians toward patient safety on patient safety outcomes in Saudi Arabian hospitals. Patient safety is at the heart of any healthcare organization, and policies and procedures are directed at facilitating the safety of patients and improving patient outcomes (5, 13).

Understanding patient safety and the role of HCPs in facilitating it is important in promoting the safety of patients and ensuring desirable outcomes (14). The manner in which nurses, physicians, and other HCPs working in the ED view patient safety is crucial. Although the advocacy for evidence-based practice has increased, the unavailability of adequate evidence on the attitude of HCPs and their implications on the outcomes of patient care may impede the development of evidence-based approaches. In this regard, research should be conducted to contribute to the evidence of the implications of HCP attitude toward patient safety, particularly in the ED of PSMMC.

### 1.1 Aim of the study

This study explored the attitude of HCPs toward patient safety in the ED of PSSMC. Specifically, it sought to achieve the following research objectives:

1. Identify the demographic characteristics of HCPs.
2. Determine the attitude of HCPs toward patient safety.
3. Determine the relationship between the safety attitude and demographic characteristics of HCPs.
4. Identify demographic factors predicting the attitude of HCPs toward patient safety.

## 2 Materials and methods

### 2.1 Research design

This quantitative study employed a cross-sectional and correlational design, selected for its effectiveness in proving and disproving assumptions. This method was also less costly and only required a short time to perform. The cross-sectional and correlation design was used to explore the relationship between patient safety attitude and demographic characteristics of HCPs in the ED of PSMMC. It was also utilized to identify demographic factors predictive of the HCPs’ attitude toward patient safety.

### 2.2 Setting

The study was conducted in the ED of PSMMC in Riyadh City, Saudi Arabia, which included areas for adults and pediatrics arranged as triage rooms, resuscitation rooms, fast track, critical care units, acute care units, and isolation rooms, with a total bed capacity of 245.

### 2.3 Population and study sample

The study targeted HCPs, including physicians, paramedics, emergency medical technicians (EMTs), and nurses. The ED was selected considering the critical state of the patients and its implications on their safety. The ED is one of the hospital units with a high incidence of medical errors (8).

### 2.4 Sample size and selection

For the sample size, this study employed the G*Power version 3.1.9.7 software, utilizing *a priori* sample size computation with given values for effect size, alpha error probability, and power. When using the software for multiple linear regression analysis with 12 predictor demographic variables, the computation with a medium effect size of 0.15, alpha error probability of 0.05, and power of 0.95 yielded a minimum sample size of 184. Hence, this study employed a convenience sampling method and obtained 202 HCPs (12 physicians, 159 nurses, and 31 paramedics and EMTs) from the ED of PSMMC, which met the required minimum sample size. The inclusion criteria involved physicians, EMTs, nurses, and paramedics who worked for the ED of PSMMC for the past 1 year or more. Meanwhile, all HCPs who did not belong to the four mentioned professions were excluded. In addition, all healthcare professionals who worked for the ED at the hospital for less than a year were excluded. Finally, HCPs who did not work under the ED were excluded.

### 2.5 Research instrument

The research questionnaire for the current study was composed of two parts. The first part was about the demographic profile or characteristics of HCPs. The second part was about the patient safety attitude of HCPs in the ED of PSMMC.

#### 2.5.1 Demographic profile/characteristics

The demographic profile of HCPs included their age, gender, marital status, nationality, educational attainment, length of experience as HCP, length of experience in the ED, certification as HCP for the ED, patient safety courses/trainings attended, number of incidents or errors encountered in the past year, and their value to patient safety. Such errors may include any accident or injury to a patient, omitted treatment, medication error, errors in transmission of a doctor’s order, errors in documentation, falls, failure to change a dressing, missed treatment, or omission of a required intervention.

#### 2.5.2 Patient safety attitude scale

The second part of the questionnaire was the Patient Safety Attitude Scale, which was adopted from the study of Durgun and Kaya (15). It was a single-page questionnaire that could be completed within 10 to 15 min. It included six domains or subscales, such as team climate with 6 items, safety climate with 7 items, job satisfaction with 5 items, stress recognition with 4 items, perception of unit management with 6 items, and working conditions with 3 items, with a total of 31 items. Each item can be answered using a five-point Likert scale, with response categories ranging from disagree strongly, disagree, neutral, agree, and agree strongly. The response categories were assigned numerical values of 1, 2, 3, 4, and 5, respectively. The scale originally demonstrated good psychometric properties and a reliability of 0.90, and thus can be used by healthcare organizations to measure the attitude of HCPs in six patient safety-related domains (16). In this study, the mean score cutoff or threshold was 3.40, which meant that any score above 3.40 was considered “positive attitude,” any score between 2.61 and 3.40 was deemed “neutral,” while a score below 2.61 was marked as “negative attitude.” In addition, the overall scale showed good reliability in the current study with Cronbach’s alpha of 0.93 as well as in its subscales for team climate (*α* = 0.78), safety climate (*α* = 0.75), job satisfaction (*α* = 0.81), stress recognition (*α* = 0.77), perception of unit management (*α* = 0.83), and working conditions (*α* = 0.79).

### 2.6 Ethical considerations

Institutional Review Board (IRB) approval was sought from the Ethical and Research Committee at King Saud University and the administration at PSMMC prior to the conduct of the study. The respondents were asked to maintain anonymity when responding to the questionnaires, and the researchers ensured that their email addresses were protected from access by any other party apart from the research team. To ensure non-sharing of the data collected, the research team eliminated any identifying information, including demographic data, from the final results and destroyed them 2 months after the research to protect the confidential information of the respondents. The respondents were expected to participate voluntarily and signed an informed consent form, which provided a summary of the study’s aim and the terms and conditions of participation. They were free to withdraw from the study at any time, without any consequences. Furthermore, they were informed that withdrawal of their participation would not put them at any harm physically and even at work. This would not affect or influence their job at the hospital.

### 2.7 Data collection procedure

After obtaining the ethics approval from the hospital, the structured questionnaire was administered to the respondents through a paper–and–pencil/pen survey. Data were collected in December 2022. All respondents were given ample time (approximately 10–15 min) to respond to the questionnaire. The questionnaire included a section on demographic information and another section with a safety attitude scale, with an included Likert scale providing a 5-point range of responses to the items, ranging from “strongly disagree” (1) to “strongly agree” (5). Negatively stated items were reversely scored prior to data analysis.

### 2.8 Data analysis

Data were analyzed using SPSS Version 23. Descriptive statistics were used for all study variables. The percentage was calculated to identify and determine the demographic characteristics of HCPs. Specifically, the following variables, such as age, number of years as HCP (in general) and as HCP in ED, number of incidents, and value of patient safety, were considered and analyzed as continuous data. Seven variables were treated as nominal scale, including gender (dichotomous), marital status (dichotomous), nationality, educational attainment, having certification as HCP in ED (dichotomous), having patient safety training (dichotomous), and profession. Weighted mean and standard deviation were calculated to measure the level of patient safety attitude of HCPs, and this variable was considered as ordinal data. Prior to conducting data analysis, the responses to negatively stated items were reverse-coded.

Meanwhile, the assessment of normality employed both the Kolmogorov–Smirnov and Shapiro–Wilk tests. The results of the normality tests indicated that the data followed a normal distribution (*p* > 0.05), with only one exception for team climate domain (Kolmogorov–Smirnov yielded *p* = 0.010 and Shapiro–Wilk yielded *p* = 0.001). Hence, the Pearson r correlation test and multiple linear regression analyses were performed to establish any relationship between patient safety attitude and demographic characteristics, as well as predictor variables. Although the patient safety attitude scale is Likert-based (ordinal), composite mean scores were treated as continuous variables, consistent with common practice in survey research. While the team climate domain did not fully meet normality assumptions, parametric analyses were retained given the large sample size and their robustness to minor deviations from normality.

Dummy variables were created for the following variables such as nationality, educational attainment, and profession prior to utilization in the linear regression model. The significance level was set at a probability value of 0.05.

## 3 Results

### 3.1 Demographic characteristics of HCPs

Table 1 shows the descriptive statistics of the demographic data of HCPs in the ED of PSMMC. The average age of the respondents was 32 years, and the majority of them were women and singles. The majority of them were Filipinos with bachelor’s degrees who worked as HCPs for an average of 8 years. Meanwhile, the respondents worked as HCPs in the ED for an average of 5 years. The majority of the respondents were certified as ED-HCPs and underwent patient safety training. They only reported an average of 1 incident or error in the ED in the past year, which might indicate high value for patients’ safety, with an average score of 9 out of 10. Finally, 79% of the respondents were nurses, 15% were paramedics and EMTs, and 6% were doctors/physicians.

**Table 1:** Demographic profile of the respondents (*n* = 202).

Socio-demographic profile	Range	Mean	Standard deviation (SD)
Age (in years)	22 to 50	31.95	5.55
Number of years as healthcare provider (HCP)	1 to 29	7.85	5.02
Number of years as an HCP in the emergency department (ED)	1 to 22	4.92	3.98
Number of incidents	0 to 10	0.85	1.72
Value to patient safety	5 to 10	9.39	1.15

	Frequency (f)	Percentage (%)
Gender		
Men	53	26.24
Women	149	73.76
Marital status		
Married	87	43.07
Single	114	56.44
Widowed	1	0.50
Nationality		
Filipino	132	65.35
Saudi	35	17.33
Other nationalities	35	17.33
Educational attainment		
Diploma	22	10.89
Bachelor	174	86.14
Master	5	2.48
Doctorate	1	0.50
Certification as HCP in ED		
Yes	174	86.14
No	28	13.86
Patient safety training		
Yes	175	86.62
No	27	13.37
Profession		
Nurse	159	78.71
Doctor	12	5.94
Paramedic and emergency medical technician (EMT)	31	15.35

### 3.2 Attitude of HCPs toward patient safety

Overall, the HCPs had positive attitude toward patient safety (mean = 3.75 out of 5), as shown in Table 2. It is also modest to note that, on average, none of the items in the attitude toward patient scale received a negative rating from the HCPs in the ED of PSMMC.

**Table 2:** Attitude of the respondents toward patient safety (*n* = 202).

Items	Mean	SD
*Team climate*	3.72	0.75
1. As a healthcare provider, my input is well received in this clinical area.	3.90	0.76
2. In this clinical area, it is difficult to speak up if I perceive a problem with patient care.	3.30	0.95
3. Disagreements in this clinical area are resolved appropriately (i.e., not who is right, but what is best for the patient).	3.69	0.81
4. I have the support I need from other personnel to care for patients.	3.75	0.77
5. It is easy for personnel in this clinical area to ask questions when there is something that they do not understand.	3.87	0.76
6. Healthcare workers here work together as a well-coordinated team.	3.85	0.89
*Safety climate*	3.87	0.70
1. I would feel safe being treated here as a patient.	3.78	0.73
2. Medical errors are handled appropriately in this clinical area.	3.96	0.78
3. I know the proper channels to direct questions regarding patient safety in this clinical area.	4.06	0.71
4. I receive appropriate feedback about my performance.	3.89	0.78
5. In this clinical area, it is difficult to discuss errors.	3.47	1.01
6. I am encouraged by my colleagues to report any patient safety concerns I may have.	3.95	0.88
7. The culture in this clinical area makes it easy to learn from the errors of others.	3.97	1.03
*Job satisfaction*	3.96	0.80
1. I like my job.	4.13	0.82
2. Working in this hospital is like being part of a large family.	3.98	0.85
3. This clinical area is a good place to work.	3.84	0.83
4. I am proud to work in this clinical area.	4.05	0.81
5. Morale in this clinical area is high.	3.82	0.78
*Stress recognition*	3.54	0.96
1. When my workload becomes excessive, my performance is impaired.	3.69	0.98
2. I am less effective at work when fatigued.	3.74	1.02
3. I am more likely to make errors in tense or hostile situations.	3.38	1.09
4. Fatigue impairs my performance during emergency situations (e.g., emergency resuscitation, seizure).	3.35	1.05
*Perception of unit management*	3.58	0.71
1. Management supports my daily efforts.	3.70	0.86
2. Management does not knowingly compromise the safety of patients.	3.50	0.88
3. Management is doing a good job.	3.61	0.79
4. Problem personnel in this clinical area are dealt with constructively by our management.	3.69	0.76
5. I get adequate, timely information about events in the hospital that might affect my work from the unit management.	3.61	0.75
6. The staffing levels in this clinical area are sufficient to handle the number of patients.	3.39	0.94
*Working Conditions*	3.80	0.78
1. This hospital does a good job of training new personnel.	3.77	0.83
2. All the necessary information for diagnostic and therapeutic decisions is routinely available to me.	3.80	0.79
3. Trainees in my discipline are adequately supervised.	3.83	0.77
*Overall patient safety attitude*	3.75	0.91

SD is the standard deviation.

In terms of the team climate, the results showed an average mean of 3.72 with a standard deviation (SD) of 0.75, which indicated that the respondents showed positive attitude toward patient safety in team climate. The item indicating HCPs agreed that their inputs were well received had the highest mean score of 3.90 (SD = 0.76), while the item indicating the respondents agreed that speaking up if they perceived a problem with patient care was difficult had the lowest mean score of 3.30 (SD = 0.95), which is below the mean score cutoff; nevertheless, this indicated neutral attitude toward patient safety.

In the context of safety climate, the item indicating that respondents knew the proper channels to direct questions regarding patient safety had the highest mean score (mean = 4.06, SD = 0.71). Conversely, the item reflecting the respondents considered that discussing errors in the ED was difficult had the lowest mean score (mean = 3.47, SD = 1.01). Nevertheless, the respondents indicated an average positive attitude toward patient safety in terms of safety climate (average mean = 3.87, SD = 0.70).

The attitude of HCPs toward patient safety, specifically in terms of job satisfaction, yielded an average mean score of 3.96 (SD = 0.80). The highest mean score was reported by HCPs agreeing that they liked their job (mean = 4.13, SD = 0.82), while the item reflecting that there was high morale within the ED received the lowest means score (mean = 3.82, SD = 0.78). Regarding stress recognition, the results indicated an average mean of 3.54 (SD = 0.96), which revealed that the respondents showed positive attitude toward patient safety. The item that received the highest reported mean score pertained to the respondents’ agreement that they were less effective at work when fatigued (mean = 3.74, SD = 1.02). This particular item was reverse-coded, implying that the HCPs were actually more effective at work, even in situations where they experienced fatigue. Meanwhile, the item that indicated fatigue impaired the respondents’ performance during emergency situations, such as resuscitation and seizure, garnered the lowest means core (mean = 3.35, SD = 1.05).

The results revealed an average mean of 3.58 (SD = 0.71), indicating that the respondents held positive attitude toward patient safety in terms of the perception of unit management. The HCPs rated with the highest mean score to the item reflecting that the management supported their daily efforts (mean = 3.70, SD = 0.86), whereas the item stating that the staffing levels in the ED were sufficient to handle the number of patients received the lowest mean score (mean = 3.39, SD = 0.94).

Finally, in the context of the working conditions domain, the findings demonstrated an average mean of 3.80 (SD = 0.78), indicating that the respondents had positive attitude toward patient safety in the ED at PSMMC. Among the three items assessed in this domain, the statement regarding trainees in the discipline that were adequately supervised received the highest means score (mean = 3.83, SD = 0.77), followed by the item reflecting that all the necessary information for diagnostic and therapeutic decisions was routinely available to the HCPs (mean = 3.80, SD = 0.79). The item that received the lowest mean score pertained to the statement indicating that the hospital did a good job of training new personnel (mean = 3.77, SD = 0.83).

### 3.3 Correlation and multiple linear regression analyses

In this study, seven correlation analyses were conducted using the Pearson r correlation test, together with seven linear regression analyses (see Table 3). In terms of team climate domain, the correlation test showed that there were three demographic variables: Nationality (*r* = −0.012, *p* = 0.014), years of experience in ED (*r* = −0.115, *p* = 0.037), and number of incidents (*r* = −0.014, *p* = 0.001) exhibited significant relationships with team climate. Meanwhile, the regression model of team climate was statistically significant [*F* (12, 187) = 2.078, *p* = 0.020], explaining approximately 34.3% of the variance (*R*^2^ = 0.118, adjusted *R*^2^ = 0.061). The model identified three demographic variables as significant predictors of team climate, including educational attainment [*β* = 0.202, *p* = 0.045, 95% confidence interval (CI) = 0.005, 0.400], years of experience in ED (*β* = −0.033, *p* = 0.031, 95% CI = −0.062, −0.003), and value of patient safety (*β* = 0.080, *p* = 0.015, 95% CI = 0.015, 0.145).

**Table 3:** Results of Pearson’s r correlation and multiple linear regression analyses (*n* = 202).

Study variables		*r*	*p*	Unstandardized coefficients		Standardized coefficients	*t*	*p*	95% confidence interval	
				*β*	*SE-b*	*Beta*			Lower limit	Upper limit
Team climate	Age	−0.036	0.470	0.000	0.011	0.004	0.034	0.973	−0.021	0.021
	Gender	−0.005	0.402	−0.112	0.100	−0.095	−1.113	0.267	−0.309	0.086
	Marital status	0.018	0.435	0.018	0.073	0.018	0.249	0.803	−0.125	0.162
	Nationality	−0.012	0.014*	−0.035	0.034	−0.095	−1.032	0.303	−0.102	0.032
	Educational attainment	0.156	0.465	0.202	0.100	0.151	2.018	0.045*	0.005	0.400
	Number of years as HCP	−0.006	0.053	0.015	0.014	0.143	1.082	0.281	−0.012	0.042
	Number of years in ED	−0.115	0.037*	−0.033	0.015	−0.252	−2.171	0.031*	−0.062	−0.003
	Certification in ED	0.127	0.097	0.107	0.116	0.072	0.919	0.359	−0.123	0.336
	Training	0.092	0.422	0.074	0.118	0.049	0.626	0.532	−0.159	0.306
	Number of incidents	−0.014	0.001***	−0.003	0.022	−0.010	−0.137	0.891	−0.047	0.041
	Value of safety	0.221	0.083	0.080	0.033	0.177	2.445	0.015*	0.015	0.145
	Profession	−0.098	0.307	−0.081	0.072	−0.116	−1.121	0.264	−0.224	0.062
Safety climate	Age	0.122	0.042*	−0.006	0.014	−0.049	−0.423	0.673	−0.034	0.022
	Gender	0.026	0.355	−0.042	0.133	−0.028	−0.317	0.751	−0.306	0.221
	Marital status	−0.079	0.133	−0.019	0.097	−0.015	−0.200	0.841	−0.211	0.172
	Nationality	0.030	0.337	−0.052	0.045	−0.108	−1.156	0.249	−0.141	0.037
	Educational attainment	0.035	0.313	−0.031	0.133	−0.017	−0.229	0.819	−0.294	0.233
	Number of years as HCP	0.209	0.001***	0.041	0.018	0.306	2.289	0.023*	0.006	0.077
	Number of years in ED	0.117	0.050*	−0.012	0.020	−0.071	−0.604	0.547	−0.052	0.027
	Certification in ED	−0.004	0.479	−0.122	0.155	−0.062	−0.786	0.433	−0.427	0.184
	Training	0.027	0.350	0.085	0.157	0.043	0.539	0.590	−0.225	0.394
	Number of incidents	−0.038	0.295	−0.030	0.029	−0.075	−1.005	0.316	−0.088	0.028
	Value of safety	0.162	0.011*	0.060	0.044	0.102	1.380	0.169	−0.026	0.146
	Profession	−0.131	0.032*	−0.175	0.096	−0.191	−1.824	0.070	−0.034	0.022
Job statisfaction	Age	0.114	0.054	0.014	0.013	0.119	1.047	0.296	−0.012	0.039
	Gender	0.023	0.373	0.016	0.122	0.011	0.130	0.896	−0.225	0.257
	Marital status	−0.046	0.258	−0.016	0.089	−0.013	−0.181	0.857	−0.191	0.159
	Nationality	−0.035	0.309	−0.037	0.041	−0.083	−0.900	0.369	−0.119	0.044
	Educational attainment	−0.003	0.484	−0.048	0.122	−0.029	−0.395	0.693	−0.289	0.193
	Number of years as HCP	0.125	0.039*	0.034	0.017	0.270	2.060	0.041*	0.001	0.067
	Number of years in ED	−0.037	0.303	−0.054	0.018	−0.340	−2.958	0.003**	−0.090	−0.018
	Certification in ED	0.100	0.079	0.014	0.142	0.007	0.096	0.923	−0.266	0.293
	Training	0.092	0.098	0.143	0.144	0.077	0.998	0.320	−0.140	0.427
	Number of incidents	0.012	0.435	0.011	0.027	0.030	0.414	0.680	−0.042	0.064
	Value of safety	0.253	0.001***	0.121	0.040	0.219	3.037	0.003**	0.042	0.200
	Profession	−0.049	0.247	−0.036	0.088	−0.042	−0.408	0.684	−0.210	0.138
Stress recognition	Age	0.003	0.482	−0.021	0.017	−0.144	−1.232	0.219	−0.054	0.013
	Gender	0.065	0.182	0.079	0.160	0.043	0.489	0.625	−0.238	0.395
	Marital status	−0.021	0.386	−0.019	0.117	−0.012	−0.163	0.871	−0.249	0.211
	Nationality	0.031	0.333	−0.015	0.054	−0.026	−0.269	0.789	−0.122	0.092
	Educational attainment	−0.040	0.287	−0.227	0.160	−0.109	−1.417	0.158	−0.544	0.089
	Number of years as HCP	0.069	0.166	0.026	0.022	0.160	1.175	0.241	−0.017	0.068
	Number of years in ED	0.051	0.237	0.004	0.024	0.020	0.170	0.865	−0.043	0.052
	Certification in ED	−0.070	0.163	−0.093	0.186	−0.040	−0.501	0.617	−0.461	0.274
	Training	−0.084	0.119	−0.144	0.189	−0.061	−0.765	0.445	−0.516	0.228
	Number of incidents	0.156	0.014*	0.076	0.035	0.164	2.162	0.032*	0.007	0.146
	Value of safety	−0.062	0.192	−0.039	0.052	−0.055	−0.736	0.463	−0.142	0.065
	Profession	−0.091	0.099	−0.121	0.116	−0.111	−1.046	0.297	−0.349	0.107
Perception of unit management	Age	−0.133	0.030*	−0.001	0.012	−0.012	−0.103	0.918	−0.024	0.022
	Gender	−0.037	0.301	−0.290	0.110	−0.223	−2.638	0.009**	−0.507	−0.073
	Marital status	0.116	0.050*	0.114	0.080	0.104	1.426	0.155	−0.044	0.271
	Nationality	0.040	0.287	−0.019	0.037	−0.047	−0.513	0.608	−0.092	0.054
	Educational attainment	0.044	0.270	0.011	0.110	0.007	0.100	0.921	−0.206	0.228
	Number of years as HCP	−0.138	0.026*	−0.004	0.015	−0.037	−0.284	0.777	−0.034	0.025
	Number of years in ED	−0.184	0.004**	−0.023	0.016	−0.163	−1.423	0.156	−0.056	0.009
	Certification in ED	0.113	0.056	0.147	0.128	0.089	1.150	0.252	−0.105	0.398
	Training	0.019	0.397	−0.012	0.129	−0.007	−0.096	0.924	−0.267	0.242
	Number of incidents	−0.010	0.446	0.009	0.024	0.028	0.384	0.701	−0.038	0.057
	Value of safety	0.183	0.005**	0.076	0.036	0.152	2.114	0.036*	0.005	0.147
	Profession	−0.165	0.010**	−0.217	0.079	−0.279	−2.735	0.007**	−0.373	−0.060
Working conditions	Age	−0.003	0.484	0.018	0.014	0.143	1.268	0.206	−0.010	0.046
	Gender	0.039	0.291	−0.082	0.134	−0.052	−0.613	0.541	−0.345	0.182
	Marital status	0.046	0.259	0.053	0.097	0.040	0.544	0.587	−0.139	0.244
	Nationality	0.062	0.192	−0.005	0.045	−0.010	−0.106	0.916	−0.094	0.084
	Educational attainment	0.124	0.040*	0.191	0.134	0.106	1.429	0.155	−0.073	0.454
	Number of years as HCP	−0.034	0.318	0.005	0.018	0.039	0.302	0.763	−0.030	0.041
	Number of years in ED	−0.156	0.014*	−0.053	0.020	−0.304	−2.645	0.009**	−0.092	−0.013
	Certification in ED	0.137	0.026*	0.220	0.155	0.110	1.420	0.157	−0.086	0.526
	Training	0.030	0.339	−0.039	0.157	−0.019	−0.246	0.806	−0.348	0.271
	Number of incidents	0.013	0.425	0.017	0.029	0.042	0.577	0.565	−0.041	0.075
	Value of safety	0.233	0.001**	0.124	0.044	0.204	2.836	0.005**	0.038	0.210
	Profession	−0.132	0.031*	−0.080	0.096	−0.085	−0.832	0.406	−0.270	0.110
Overall attitude	Age	−0.006	0.465	0.004	0.010	0.045	0.396	0.693	−0.016	0.023
	Gender	0.005	0.472	−0.133	0.093	−0.121	−1.428	0.155	−0.317	0.051
	Marital status	0.032	0.329	0.044	0.068	0.047	0.647	0.519	−0.090	0.178
	Nationality	0.028	0.348	−0.028	0.032	−0.081	−0.883	0.378	−0.090	0.034
	Educational attainment	0.082	0.124	0.056	0.093	0.045	0.600	0.549	−0.128	0.240
	Number of years as HCP	0.013	0.430	0.015	0.013	0.150	1.149	0.252	−0.010	0.040
	Number of years in ED	−0.111	0.059	−0.033	0.014	−0.272	−2.367	0.019*	−0.061	−0.006
	Certification in ED	0.121	0.044*	0.085	0.108	0.061	0.788	0.432	−0.128	0.299
	Training	0.057	0.211	0.040	0.110	0.028	0.362	0.718	−0.177	0.256
	Number of incidents	−0.009	0.447	0.002	0.021	0.008	0.114	0.909	−0.038	0.043
	Value of safety	0.260	0.001**	0.090	0.031	0.211	2.935	0.004**	0.029	0.150
	Profession	−0.155	0.014*	−0.134	0.067	−0.204	−1.996	0.047*	−0.267	−0.002

The dependent variable was the average mean of the six domains and the overall mean of the patient safety attitude.

*r* is the Pearson r correlation value; *p* is the *p*-value; *β* is the unstandardized coefficients; SE-b is the standard error; *t* is the *t*-value. *Significance level at 0.05. **Significance level at 0.01. ***Significance level at 0.001.

In terms of safety climate domain, the correlation test indicated that five demographic variables, namely, age (*r* = 0.122, *p* = 0.042), years of experience as HCP (*r* = 0.209, *p* = 0.001), years of experience in ED (*r* = 0.117, *p* = 0.050), value of patient safety (*r* = 0.162, *p* = 0.011), and profession (*r* = −0.131, *p* = 0.032), revealed significant relationships with safety climate. In the meantime, the regression model regarding safety climate resulted in a statistically insignificant finding [*F* (12, 187) = 1.582, *p* = 0.100], accounting for approximately 30.4% of the variance (*R*^2^ = 0.092, adjusted *R*^2^ = 0.034). Nevertheless, the model identified a single demographic variable that maintained its individual significance regarding safety climate, particularly the years of experience as HCP (*β* = 0.041, *p* = 0.023, 95% CI = 0.006, 0.077). The regression model for safety climate was not significant (*p* = 0.100); therefore, the results for individual predictors should be interpreted with caution.

Regarding the job satisfaction domain, the correlation test resulted in having two demographic variables: years of experience as HCP (*r* = 0.125, *p* = 0.039) and value of patient safety (*r* = 0.253, *p* = 0.001) exhibiting significant relationships with job satisfaction. Meanwhile, the regression model of job satisfaction was statistically significant [*F* (12, 187) = 2.311, *p* = 0.009], explaining approximately 35.9% of the variance (*R*^2^ = 0.129, adjusted *R*^2^ = 0.073). The model identified three demographic variables as significant predictors of job satisfaction, including years of experience as HCP (*β* = 0.034, *p* = 0.041, 95% CI = 0.001, 0.067), years of experience in ED (*β* = −0.054, *p* = 0.003, 95% CI = −0.090, −0.018), and value of patient safety (*β* = 0.121, *p* = 0.003, 95% CI = 0.042, 0.200).

For the stress recognition domain, both correlation and linear regression tests indicated that a single demographic variable, particularly the number of incidents, showed a significant relationship (*r* = 0.156, *p* = 0.014) and also served as a predictor variable (*β* = 0.076, *p* = 0.032, 95% CI = 0.007, 0.146) for stress recognition. Although the regression model for stress recognition was found to be statistically insignificant [*F* (12, 187) = 1.088, *p* = 0.372], explaining approximately 25.5% of the variance (*R*^2^ = 0.065, adjusted *R*^2^ = 0.005), the number of incidents retained its individual significance as a predictor of stress recognition. The regression model for stress recognition was not significant (*p* = 0.372); therefore, the results for individual predictors should be interpreted with caution.

With regard to perception of unit management domain, the correlation test showed that six demographic variables, namely, age (*r* = −0.133, *p* = 0.030), marital status (*r* = 0.116, *p* = 0.050), years of experience as HCP (*r* = −0.138, *p* = 0.026), years of experience in ED (*r* = −0.184, *p* = 0.004), value of patient safety (*r* = 0.183, *p* = 0.005), and profession (*r* = −0.165, *p* = 0.010), revealed significant correlations with perception of unit management. Meanwhile, the regression model of perception of unit management was statistically significant [*F* (12, 187) = 2.508, *p* = 0.004], explaining approximately 37.2% of the variance (*R*^2^ = 0.139, adjusted *R*^2^ = 0.083). The model identified three demographic variables as significant predictors of perception of unit management, including gender (*β* = −0.290, *p* = 0.009, 95% CI = −0.507, −0.073), value of patient safety (*β* = 0.076, *p* = 0.036, 95% CI = 0.005, 0.147), and profession (*β* = −0.217, *p* = 0.007, 95% CI = −0.373, −0.060).

For the working conditions domain, the correlation test showed that five demographic variables, namely, educational attainment (*r* = 0.124, *p* = 0.040), years of experience in ED (*r* = −0.156, *p* = 0.014), certification in ED (*r* = 0.137, *p* = 0.026), value of patient safety (*r* = 0.233, *p* = 0.001), and profession (*r* = −0.132, *p* = 0.031), revealed significant correlations with working conditions. Meanwhile, the regression model of working conditions was statistically significant (*F* [12, 187] = 2.368, *p* = 0.007), explaining approximately 36.3% of the variance (*R*^2^ = 0.132, adjusted *R*^2^ = 0.076). The model revealed two demographic variables as significant predictors of working conditions, including years of experience in ED (*β* = −0.053, *p* = 0.009, 95% CI = −0.092, −0.013) and value of patient safety (*β* = 0.124, *p* = 0.005, 95% CI = 0.038, 0.210).

Finally, the overall attitude toward patient safety among HCPs exhibited significant relationships with three demographic variables: certification in ED (*r* = 0.121, *p* = 0.044), value of patient safety (*r* = 0.260, *p* = 0.001), and profession (*r* = −0.155, *p* = 0.014). There are also three demographic variables, namely, years of experience in ED (*β* = −0.033, *p* = 0.019, 95% CI = −0.061, −0.006), value of patient safety (*β* = 0.090, *p* = 0.004, 95% CI = 0.029, 0.150), and profession (*β* = −0.134, *p* = 0.047, 95% CI = −0.267, −0.002), that were identified as predictors of the overall patient safety attitude of the HCPs. The regression model for the overall patient safety attitude yielded statistically significant results [*F* (12, 187) = 2.323, *p* = 0.009], explaining approximately 36.0% of the variance (*R*^2^ = 0.130, adjusted *R*^2^ = 0.074).

## 4 Discussion

This study aimed to explore the attitude of HCPs toward patient safety in the ED of PSSMC and to identify demographic factors predicting the HCPs’ attitude. Patient safety is defined as prevention and avoidance of patient injuries or adverse events caused by healthcare delivery (17). The increase in number of patient safety incidents poses a challenge for hospital management. Studying the attitude of HCPs toward patient safety is important to deal with these situations (1, 18). The current findings are contrary to reports in the previous study (18), where the participants only reported an average of 1 incident in the previous year. In the present work, the respondents received certification as ED-HCPs, and most of them underwent patient safety training, leading to their high value for patients’ safety, with an average score of 9 out of 10. Safety attitude was also investigated in different countries and hospital departments (19) to improve patient safety culture in hospitals (20, 21).

Overall, the HCPs reported a positive attitude toward patient safety in the current study, consistent with previous research works. For instance, a study in a neighboring Arab country in Türkiye aimed to identify the attitude of nurses toward patient safety in the ED of selected third-level hospitals and found that the nurses generally had a positive attitude (15). A cross-sectional study about the attitude of doctors and nurses to patient safety and errors in medical practice was conducted with a convenience sample of 424 nurses and 150 physicians working for at least 6 months in the studied hospitals; the participants had moderately positive attitude toward patient safety (22). By contrast, the findings of another study revealed that nurses and doctors had less than positive attitude toward patient safety (10). Lisbon et al. (23) reported that the safety attitude of physicians and nurses from two EDs was generally less than positive even after administering an intervention related to team building. In attaining high attitude toward patient safety, approximately 75% of the participants reported a positive attitude in a previous study of Asem, Sabry, and Elfar (17); the value was higher than the reported 50% in a previous study in Saudi Arabia (24). In general, HCPs had positive attitude to patient safety (18). Similarly, high positive attitude was reported in Italy, with a value of 100% on different items (25). In another study, the respondents’ attitude toward patient safety education was also positive (26). In another qualitative study among Egyptian nurses working in the pediatric intensive care units, the participants reported having positive attitude toward patient safety culture and viewed patient safety as an important aspect of patient care quality and a main concern to the hospital (27). Conversely, a previous study in Jordan assessed the attitude and knowledge of 904 HCPs with regard to the safe use of medications during breastfeeding; HCPs were found to have variable attitude regarding patient safety (28). Another study in Türkiye revealed that the attitude toward patient safety among staff nurses in the cardiology and cardiovascular surgery units was not at a particularly satisfactory level (29). The attitude of staff nurses working in the ED in Türkiye was only at an average level (15). Overall, the safety attitude of ED health staff is generally low, especially on management support and among nurses compared with doctors. A similar study in Saudi Arabia, where doctors and nurses’ attitude toward patient safety was investigated in the ED of two hospitals, the results showed that nurses and doctors generally had less than positive safety attitude (10).

In terms of team climate, the HCPs reported to have positive attitude toward patient safety. Although the respondents agreed that speaking up if they perceived a problem with patient care in the ED was difficult, they also stated that their inputs were well received, disagreements were resolved appropriately, and they had the support they needed from other personnel to care for patients. Furthermore, they could easily ask questions to personnel for the things that they did not understand and that HCPs in the ED worked together as a well-coordinated team. According to a systematic review about safety attitude in the ED, teamwork is one of the aspects that promotes positive attitude (10). Human resource issues, such as teamwork and management support, were related to lower safety attitudes among hospital staff. Interventions aimed at improving teamwork and management support are likely to have a positive impact on safety attitudes (10). Another study that used a quantitative repeated measures design and a team building intervention reported post-intervention success because the safety culture attitude of the participants demonstrated improved communication and teamwork (23). Furthermore, the teamwork climate score was answered high or very high by the participants in the study conducted by Gadallah et al. (30). Accordingly, teamwork is one of the aspects central to positive attitude, and teamwork training can improve attitude toward patient safety (31). Laal et al. (32) reported that teamwork within the department had the most significant correlation with patient safety culture. Another review study that employed qualitative design reported that teamwork and team support were critical to enhance patient safety and that positive safety attitude was associated with teamwork (33). Other researchers have explored the attitude of HCPs regarding patient safety and found that the overall safety attitude was positive; however, they obtained lower scores with teamwork climate (34). A literature review revealed that the safety attitude of ED healthcare staff was generally low, especially teamwork among nurses when compared with doctors; on multidimensional safety attitude scales, teamwork was often rated as relatively low (19).

In terms of safety climate, the HCPs in the ED at PSMMC had positive attitude toward patient safety. However, the majority of the HCPs agreed that discussing errors in the ED was difficult. Nevertheless, they agreed that the patient would feel safe being treated in the ED, medical errors were handled appropriately, and HCPs knew the proper channels to direct questions regarding patient safety and received appropriate feedback about their performance. Furthermore, the HCPs were encouraged by their colleagues to report any patient’s safety concerns that they might have, and the culture in the ED made it easy for them to learn from the errors of others. Gadallah et al. (30) reported that the safety climate score was high or very high among the participants. When the mean scores obtained among the participants were compared with the data of another study, the midwife participants had the highest score in the subscales of safety climate (35). However, the results of a previous study contradicted the current study, where only 39 physicians had a positive attitude toward safety climate, and more than 50% of the physicians and nurses surveyed were dissatisfied with their jobs (18).

In terms of job satisfaction, the HCPs in the ED at PSMMC had positive attitude toward patient safety. The majority of the HCPs agreed to the statements that they liked their job, working in the hospital was like being part of a large family, the ED was a good place to work and had a high morale, and they were proud to work in the ED of PSMMC. Similarly, a previous study reported job satisfaction among nurses and physicians (10). Positive safety attitude was related to the levels of job satisfaction among respondents (18). However, another work found that the overall safety attitude was positive, although a safety attitude area was self-evaluated as low in job satisfaction among HCPs (34). The job satisfaction score was answered inadequate by more than 50% of the participants (30).

For stress recognition, the HCPs in the ED of PSMMC revealed that they had positive attitude toward patient safety. The majority of the respondents agreed that when their workload became excessive, their performance was impaired, and they were less effective at work when fatigued. The majority of the respondents were neutral in the statements that they were more likely to make errors in tense or hostile situations, and fatigue impaired their performance during emergency situations, such as resuscitation and seizure. By contrast, the study participants including nurses and doctors had less than positive attitude toward patient safety (10). Stress recognition score was answered few or very few and inadequate among study participants in the study conducted by Gadallah et al. (30).

In terms of perception of unit management, the HCPs in the ED of PSMMC had positive attitude toward patient safety. The majority of the respondents agreed that the management supported their daily efforts, did not knowingly compromise the safety of patients, and was doing a good job. Moreover, problem personnel were dealt with constructively by the management, and the staffing levels in the ED were sufficient to handle the number of patients. The respondents also had adequate, timely information about events in the hospital that might affect their work. In the previous literature, management support was central to positive safety attitude (19). Similarly, the perception of unit management score was answered high or very high by 58% of the participants in the study of Gadallah et al. (30). By contrast, more than 50% of nurses and doctors had less than positive attitude toward patient safety, with mean scores on perceptions of hospital unit management (10).

For the working conditions, the HCPs in the ED of PSMMC revealed that they also had positive attitude toward patient safety. The majority of the respondents agreed that the hospital did a good job of training new personnel, trainees in the discipline were adequately supervised, and all the necessary information for diagnostic and therapeutic decisions was routinely available to the HCPs. By contrast, the participants including physicians and nurses reported that the hospital working conditions were less than positive but not more negative compared with the other dimensions of patient safety attitude (10). The working condition score was answered inadequate by the majority of the respondents in the study of Gadallah et al. (30).

This study explored the relationships between the demographic characteristics of HCPs and their overall attitude toward patient safety and across the six domains of team climate, safety climate, job satisfaction, stress recognition, perception of unit management, and working conditions. The relationships between the demographic characteristics of HCPs and their attitude toward patient safety were comparable with reports in previous studies. Positive safety attitude was associated with communication and management support as well as improved management of the ED and the presence of an ED safety committee (33). The findings were contrary to the results of the following studies. According to Brasaite et al. (18), the respondents’ old age was associated with how they evaluated their job satisfaction as well as with other dimensions including teamwork climate, safety climate, and perception of management. In addition, another study in Jordan found that age and having knowledge on patient safety during continuing educational training were predictors of patient safety attitude among primary healthcare nurses (36). Moreover, the respondents’ profession, working unit, length of work experience, information received about patient safety during education, further education, and working shifts were associated with several safety attitude areas (18). The experience of the study participants had a positive impact on patient safety, particularly where nurses with experience showed expected results, used evidence efficiently, and developed critical thinking skills (15). According to Durgun and Kaya (15), despite the varying results of studies on the dimensions of patient safety, the professional experience of nurses was expected to have a positive effect. Rosseter (37) reported that the educational level of nurses was correlated with the quality of patient care, and decreased interventions endangered patient safety. An inter-correlational data showed the projected moderate relationships among the dimensions; the stress recognition dimension was generally not correlated with any of the other dimensions (10). However, a recent systematic review showed that having positive safety attitude in the hospital setting as well as in nursing unit levels resulted in lesser reports of adverse patient outcomes due to positive teamwork climate (38).

Furthermore, research evidence indicates that a positive safety attitude among HCPs, including nurses, is significantly associated with a decrease in key patient outcomes such as falls, healthcare-associated infections, medication errors, and pressure injuries (38). Thus, exploring the attitude of HCPs toward patient safety and identifying the demographic factors that predict their patient safety attitude holds considerable significance to any healthcare organization. The findings of the current study contribute to the existing body literature regarding demographic factors that significantly influence the attitude of HCPs toward patient safety. Among these predictors are gender, educational attainment, length of experience, number of incidents reported, value of patient safety, and profession. In particular, educational attainment was significantly associated with teamwork climate, suggesting that HCPs with higher education levels may feel more empowered to contribute to team-based safety practices. Similarly, years of ED experience negatively predicted some domains, which may reflect increased exposure to stressful or resource-limited situations. These factors predicting HCPs’ attitude toward patient safety align with findings from previous studies both globally and within the Saudi Arabian context. For example, continuing education that encompasses patient safety topics has been reported as a significant predictor of safety attitude score among Jordanian nurses working healthcare centers (36). Moreover, several reports have highlighted the importance of providing and ensuring education and training on patient safety to HCPs to enhance safety culture within healthcare settings, as evidenced by previous studies conducted in Malaysia (39), the Republic of Korea (40), and the United States (41). In addition, the number of incidents reported by the HCPs has also shown to significantly predict HCPs’ attitude toward patient safety. This finding is comparable to a previous study indicating that majority of the Saudi nurses did not report any incidents over the past year (42). Another noteworthy finding pertains to value of patient safety as a predictor of HCPs’ attitude toward patient safety. This finding is similar to a recent study conducted among emergency nurses in China (43). Fu et al. (43) concluded that emergency nurses’ safety value indirectly influenced their patient safety competency through safety attitude.

This study has limitations. The self-report patient safety attitude and the use of convenience sampling method might have some degree of bias when the HCPs answered the survey. The current study was undertaken among HCPs working in the ED in a tertiary military hospital in Riyadh that provided services for all health disciplines and particularly among military personnel as well as their family and relatives; as such, the results cannot be applied to all HCPs in the ED. Another limitation is the low number of HCPs as respondents, where the findings might have been influenced by the imbalanced or inadequate representation of the survey groups based on the profession of HCPs. Specifically, there were 159 nurses (78.71%) out of the total 202 respondents, the sample was highly skewed toward nurses, whereas only 12 doctors (5.94%) and 31 paramedics and EMTs (15.35%) participated in the study. This limited the study’s ability to compare attitude toward patient safety across profession groups, due to their underrepresentation doctors, paramedics, and EMTs. The findings should be treated with caution because they may not be representative of the perceived attitude toward patient safety among doctors, paramedics, and EMTs and may not be applicable to the other EDs in other healthcare settings in Saudi Arabia, including privately owned healthcare organizations. Hence, these limitations hinder generalizability of the study findings.

## 5 Conclusion and implications

The overall attitude toward patient safety of HCPs in the ED of PSMMC was positive and in all the six dimensions tested; however, some patient safety issues should be resolved, including difficulty in speaking up of a perceived problem with patient care as well as in discussing errors in the ED at PSMMC. The implications and contributions of the study to healthcare practice must be highlighted to resolve the existing patient safety issues in the ED at PSMMC. For instance, healthcare institutions and the ED may use data in this study to establish safe attitude among HCPs for the benefit of patients seeking help and to train HCPs to become more informed and aware of the importance of positive patient safety attitude. The administrators and policymakers in the ED of PSMMC must work together to consider the establishment of training programs geared to change some patient safety issues reported by the HCPs and to develop patient safety attitude in different approaches for all different professions, age groups, and nationalities. In addition, training programs should target gaps such as communication skills to encourage speaking up and error reporting. Interventions should also take into account profession and years of ED experience, as these emerged as predictors of safety attitudes. Training programs for all HCPs, along with strong management support, are important to improve and promote positive patient attitudes and performance related to patient safety. Finally, strategies and policies should be developed to promote a positive patient safety culture among the HCPs in the ED.

## 6 Recommendations

Recommendations are highlighted in terms of the patient safety issues in the dimension of team climate, where the respondents reported some degree of difficulty in speaking up if they perceived a problem with patient care. Human resource and communication issues, such as this, require interventions to improve factors that would likely affect positively the safety attitude of HCPs. Additional recommendations are suggested for solving safety climate issues, where the majority of the respondents reported difficulty in discussing errors in the ED. The inevitability of medical errors, as well as their impact and learning from them, is an essential part in providing the necessary training and continuing education to HCPs to promote positive patient safety attitude. The HCPs were reported to have differences in patient safety attitude in some dimensions of patient safety; therefore, training needs to involve everyone to create a shared vision for patient safety that can be attained through collaborative and instructive workshops. Error reporting systems that are non-punitive should be designed. Further studies with a larger sample size are needed to involve ED-HCPs in other hospitals in different regions in Saudi Arabia to establish the generalizability of the findings on the attitude of HCPs toward patient safety.

## Data Availability

The raw data supporting the conclusions of this article will be made available by the authors, without undue reservation.
